# Analysis of the Atmospheric Water Budget for Elucidating the Spatial Scale of Precipitation Changes Under Climate Change

**DOI:** 10.1029/2019GL084173

**Published:** 2019-09-02

**Authors:** Guy Dagan, Philip Stier, Duncan Watson‐Parris

**Affiliations:** ^1^ Atmospheric, Oceanic and Planetary Physics, Department of Physics University of Oxford Oxford UK

**Keywords:** precipitation, climate change, spatial scales, evaporation

## Abstract

Global mean precipitation changes due to climate change were previously shown to be relatively small and well constrained by the energy budget. However, local precipitation changes can be much more significant. In this paper we propose that for large enough scales, for which the water budget is closed (precipitation [*P*] roughly equals evaporation [*E*]), changes in *P* approach the small global mean value. However, for smaller scales, for which *P* and *E* are not necessarily equal and convergence of water vapor still plays a role, changes in *P* could be much larger due to dynamical contributions. Using 40 years of two reanalysis data sets, 39 Coupled Model Intercomparison Project Phase 5 (CMIP5) models and additional numerical simulations, we identify the scale of transition in the importance of the different terms in the water budget to precipitation to be ~3,500–4,000 km and demonstrate its relation to the spatial scale of precipitation changes under climate change.

## Key Points

- The present‐day characteristic scale of the hydrological cycle predicts the spatial scale of precipitation changes under climate change
- Using reanalysis data sets and CMIP5 models, we identify the characteristic scale of the hydrological cycle to be ~3,500–4,000 km
- These results suggest that changes in precipitation on the continental scale could be much larger than the global mean change

## 1 Introduction

Under climate change, in addition to the expected changes in surface temperature, precipitation is also expected to change, with potentially significant implications for society. Based on the Clausius‐Clapeyron relation, a warmer atmosphere leads to higher water vapor content, increasing by approximately 7% for each 1 K increase. However, the global mean precipitation response to warming is predicted to be lower (2–3%/K) than the expected by the Clausius‐Clapeyron relation (Allen & Ingram, 2002; Andrews et al., 2010; Andrews & Forster, 2010; Held & Soden, 2006). This relatively small precipitation response is consistent with the concept of an energetic control of precipitation, which states that precipitation must change in such a way that the atmospheric energy budget remains in balance (Allen & Ingram, 2002; O'Gorman et al., 2012; Pendergrass & Hartmann, 2014). Different climate forcing agents (such as different greenhouse gases and different aerosol species) affect the atmospheric energy budget and hence the precipitation differently (Dagan et al., 2019; Hodnebrog et al., 2016; Liu et al., 2018; Mitchell et al., 1987; Muller & O'Gorman, 2011; Myhre et al., 2017; O'Gorman et al., 2012; Richardson et al., 2018; Samset et al., 2016). However, on a global scale, precipitation changes due to any driver must be constrained by the energy budget.

The fact that the global mean precipitation changes are relatively small and constrained by the energy budget does not apply to changes on small temporal and spatial scales (Bony et al., 2013; Kendon et al., 2008; Lenderink & Van Meijgaard, 2008; O'Gorman, 2015). Even on long temporal scales (under equilibrium temperature response to radiative forcing), the precipitation changes on a local scale could be much larger/smaller than the global mean. It was previously proposed that the magnitude of the balance of precipitation and evaporation would increase under global warming, maintain its spatial patterns, and thus driving the local changes in the hydrological cycle (the “wet gets wetter, dry gets drier” paradigm; Held & Soden, 2006). For climatological time scales, for which both the seasonal and interannual variability in water vapor amount in the atmosphere (represented by the water vapor storage term) average out, the water budget is determined by a balance between precipitation (*P*), evaporation (*E*), and divergence of water vapor (div (*q*_*v*_); Brown & Kummerow, 2014; Newman et al., 2012; Peixoto & Oort, 1992; Trenberth et al., 2011):

(1) $$P=E-\mathrm{div}\;\left(q_{v}\right)$$

The divergence of water vapor becomes inefficient with increasing spatial scales and vanishes on the global scale. Based on this argument and equation (1), it is predicted that local changes in precipitation (due to any forcing) would be driven by changes in evaporation or water vapor divergence. The latter can be induced by both changes in atmospheric circulation, driving changes in air mass divergence, or water vapor capacity, driving changes in div (*q*_*v*_), even for a given air mass divergence (Mitchell et al., 1987). We note that changes of the dynamics/thermodynamics of the atmosphere that would change the advection of water vapor without causing any change to the divergent term or to the evaporation will not directly affect the precipitation changes.

On a large spatial scale, once the divergence term becomes small enough, precipitation changes would be equivalent to the evaporation changes. In equilibrium, both precipitation and evaporation are strongly constrained by the energy budget and are expected to be small (2–3%/K; Allen & Ingram, 2002). This implies the existence of a shift in the relative importance of the different water budget terms for precipitation changes. The objective of this study is to identify this water budget breakdown scale and to examine whether indeed it constrains the scale of changes in precipitation under climate change.

The above argument can also be put in a more commonly used perspective of thermodynamics vs. dynamics contributions to changes in precipitation (which has some similarities but is not identical to the water budget perspective presented above; Allen & Ingram, 2002; Bony et al., 2013; Ma & Xie, 2013; O'Gorman, 2015; Pfahl et al., 2017; Sato et al., 2017; Weller et al., 2019)). It is argued that the main contributions to precipitation changes are changes in air temperature and hence in water vapor amount in the atmosphere (thermodynamics contribution), and changes in the atmospheric circulation (dynamic contribution). On increasingly large to global scales, the dynamic contribution vanishes and the thermodynamics contribution dominates. However, on smaller scales the dynamic contribution can dominate (Chadwick et al., 2013; Weller et al., 2019). This, again, implies the existence of a breakdown scale of the causes deriving precipitation changes. Our aim is to identify on what spatial scales one should expect a large dynamic contribution (or large contribution from changes in div (*q*_*v*_)) and on what spatial scale thermodynamics (or the energy budget) constrain the changes in precipitation.

## 2 Methods

In this study we use CMIP5 (phase 5 of the Coupled Model Intercomparison Project) data (Taylor et al., 2012), ECMWF (European Centre for Medium‐Range Weather Forecasts) Era‐Interim reanalysis data (Dee et al., 2011), NCEP (National Centers for Environmental Prediction) Department of Energy (DOE) 2 reanalysis data (Kalnay et al., 1996), and simulations conducted with the ICON (icosahedral nonhydrostatic) model (Crueger et al., 2018; Giorgetta et al., 2018; Zängl et al., 2015) as described below.

### 2.1 CMIP5 Data

Precipitation and evaporation fields from 39 CMIP5 models (listed in Table S1 in the supporting information) are used from two scenarios: historical and RCP8.5 (Representative Concentration Pathway 8.5; Riahi et al., 2011) simulations. For each scenario, average fields over 20 years are used. Form the historical runs we use the last 20 years of the 20th century. From the RCP8.5 runs we use the last 20 years of the 21st century. The scale in which the water budget is locally closed (precipitation minus evaporation [*P* − *E*] close to 0) is determined based on the historical simulations (and compared to the scale from the RCP8.5 simulations), while changes in precipitation (*δP*) and evaporation (*δE*) are determined based on the difference between the RCP8.5 and the historical runs. In these simulations, *δP* and *δE* are forced by a combination of greenhouse gases, aerosol and land use changes. All data are remapped to 1°×1° resolution.

### 2.2 Reanalysis Data

While CMIP data are entirely model based, reanalysis data provide an observationally constrained dataset of the hydrological cycle. We use 40 years (1979–2018) of reanalysis data from ECMWF Era‐Interim and NCEP‐DOE 2. The spatial resolution of the *P* and *E* fields from the ECMWF data is 1°×1°, while from NCEP it is T63 (less than 2°).

### 2.3 ICON Simulations

To examine the effect of land on the spatial scales in the hydrological cycle, we use the ICON model. Two different simulations are conducted: one, following the AMIP protocol (Gates, 1992), with land and prescribed sea surface temperature (SST—will be referred to hereafter as ICON‐AMIP) and another without land (aqua‐planet; Wan et al., 2013) and prescribed SST (will be referred to hereafter as ICON‐aqua). For each configuration (AMIP or aqua‐planet) 10 years of simulations are conducted. The simulations are conducted with 47 vertical levels. The ICON grid R2B04 is used, which has an effective resolution of 157.8 km (Zängl et al., 2015).

## 3 Results

**Figure 1:**
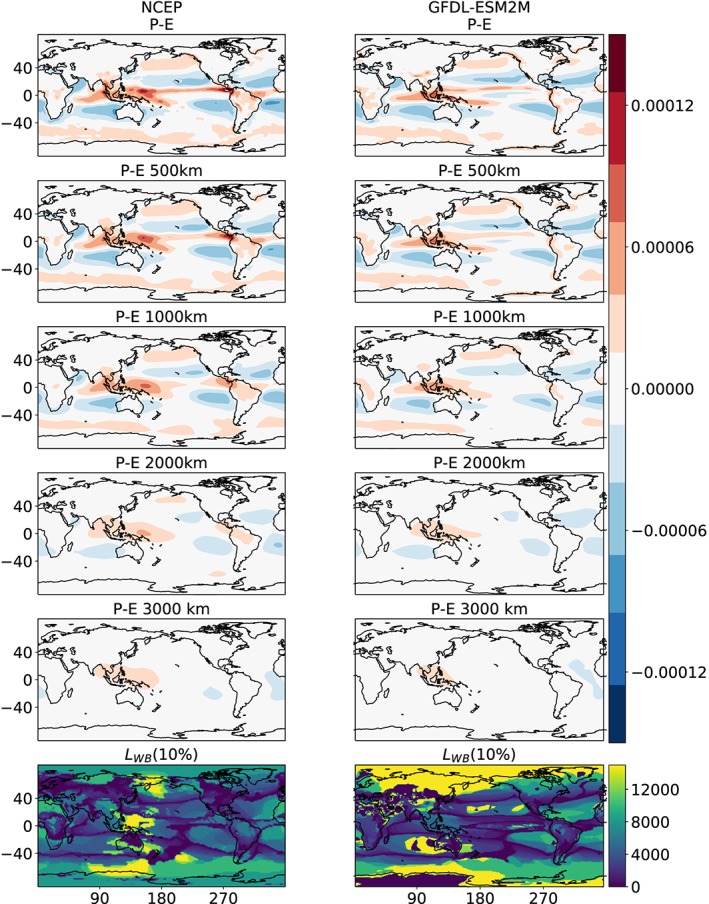
Precipitation minus evaporation (*P* − *E* [kg/s/m^2^]) at different spatial scales. The top row shows the native model resolution; the subsequent rows are averaged over a circle centered at each grid point with the given radius indicated in the title. The lower row presents the scale [km] for which the water budget is closed to within 10%, that is, (*P* − *E*)/*P* < 0.1 − *L*_*WB*_(10%). The left column presents the National Centers for Environmental Prediction (NCEP) reanalysis data, while the right column presents an example of one model from the Coupled Model Intercomparison Project Phase 5 (CMIP5) historical runs (Geophysical Fluid Dynamics Laboratory Earth System Model with Modular Ocean Model 4 component [GFDL‐ESM 2M]; Dunne et al., 2012).

Figure 1 presents the precipitation minus evaporation (*P* − *E*) fields averaged over different spatial scales from one reanalysis data set (NCEP) and one of the CMIP5 models (Geophysical Fluid Dynamics Laboratory Earth System Model with Modular Ocean Model 4 component [GFDL‐ESM 2M]; Dunne et al., 2012) as an example. At the native resolution, both the reanalysis and the CMIP5 model show a similar pattern of net *P* at the Intertropical Converge Zone, net *E* in the subtropics (which is more dominant in the eastern parts of the oceans where lower SSTs are found), and net *P* at midlatitude and high latitude. Averaging over increasingly larger scales, this pattern becomes weaker and almost completely vanishes at 3,000 km. In addition, the lower panels in Figure 1 present the scales for which the water budget is locally closed to within 10% (*L*_*WB*_[10%]), that is:

(2) $$\left(P-E\right)/P<0.1.$$

It demonstrates, as expected, that at the centre of a region of negative/positive *P* − *E* the scale needed to average over for closure of the water budget is larger, while near a transition between net *P* to net *E*, *L*_*WB*_ is lower. Hence, the emerging pattern of *L*_*WB*_ reproduces the features in the eastern parts of the subtropical oceans and a sharp transition around ±40°. At high latitudes, beyond 40°, the small net precipitation is driven by a water vapor supply from lower latitudes; consequently, *L*_*WB*_ is generally larger. Hereafter, we will focus on the region between −40 and 40°. In addition, we note that using a specific threshold (in this case 10%) may generate a sharp transition of scales at specific locations.

**Figure 2:**
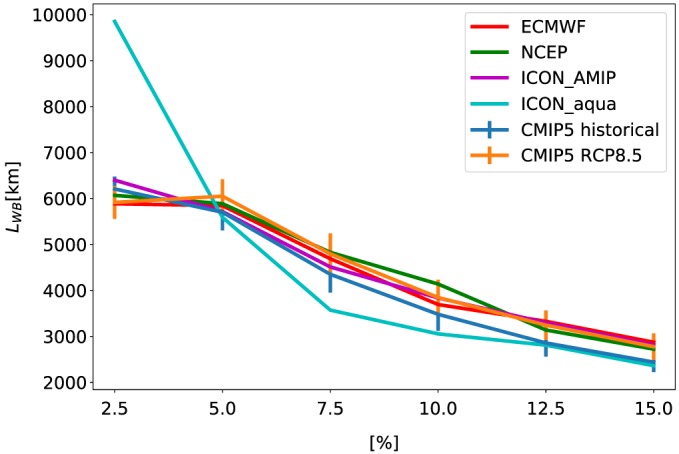
The spatial scale for local water budget closure (*L*_*WB*_—for which precipitation roughly equals evaporation) as a function of the degree of closure (i.e., the level of similarity between *P* and *E*) from the different models and reanalysis data sets. The different data sets presented here are Coupled Model Intercomparison Project Phase 5 (CMIP5) historical and Representative Concentration Pathway 8.5(RCP8.5) simulations, reanalysis data sets of European Centre for Medium‐Range Weather Forecasts (ECMWF) and National Centers for Environmental Prediction (NCEP), and additional simulations using the icosahedral nonhydrostatic (ICON) model with and without land (ICON‐AMIP and ICON‐aqua, respectively). The vertical lines in the CMIP5 curves represent the standard deviation of the 39 different models.

Figure 1 demonstrates that the water budget is locally closed once averaged radially over a few 1,000 km (74% of the precipitation in our region of interest [−40 to 40°] falls in a region in which the local water budget is closed to within 10% on 4,000‐km scale). However, the exact value depends on the degree of closure, that is, to within what percent *P* and *E* are similar (a perfect *P*=*E* is achieved only on the global scale). Figure 2 presents the mean (over −40 to 40° latitudes) scale at which the water budget is locally close (*L*_*WB*_) for different degrees of closure for all the different data sets and models. All different models and reanalysis data sets demonstrate a monotonic decrease in *L*_*WB*_ as the level of closer becomes larger and (except the ICON_aqua) are within ~20% from one another (for a given degree of closure). Figure 2 also demonstrates that *L*_*WB*_ does not significantly change with climate change (i.e., similar trends for the historical and RCP8.5 simulations). The ICON_aqua simulation demonstrates a larger sensitivity of *L*_*WB*_ to the level of closure. Since the patterns of *P* − *E* in the aqua‐planet simulation are zonally symmetric (no emerging features at the east parts of the oceans; see Figure 1), it requires larger spatial scales to get to a strict water budget closure (e.g., to within 2.5%). However, as the zonal stripes of net *P* and net *E* are located one next to the other, large fraction of the globe requires only relatively small spatial scale averaging to get to a loose closure (e.g., to within 15%). Figure 2 presents the mean scale (*L*_*WB*_) between −40 to 40° latitudes. However, as can be seen from Figure 1, there exist spatial variations in this scale. Figure S1 in the supporting information presents the average scale over land and ocean separately, as well as the average scale including higher latitudes. In addition, we note that averaging separately in the zonal and meridional directions (rather than radially) will result in a different length scale, as, for example, at latitudes of net precipitation (deep tropics or high latitudes) or at latitudes of net evaporation (subtropics) zonal averaging will not result in a closure of the water budget even once averaged over all longitudes.

**Figure 3:**
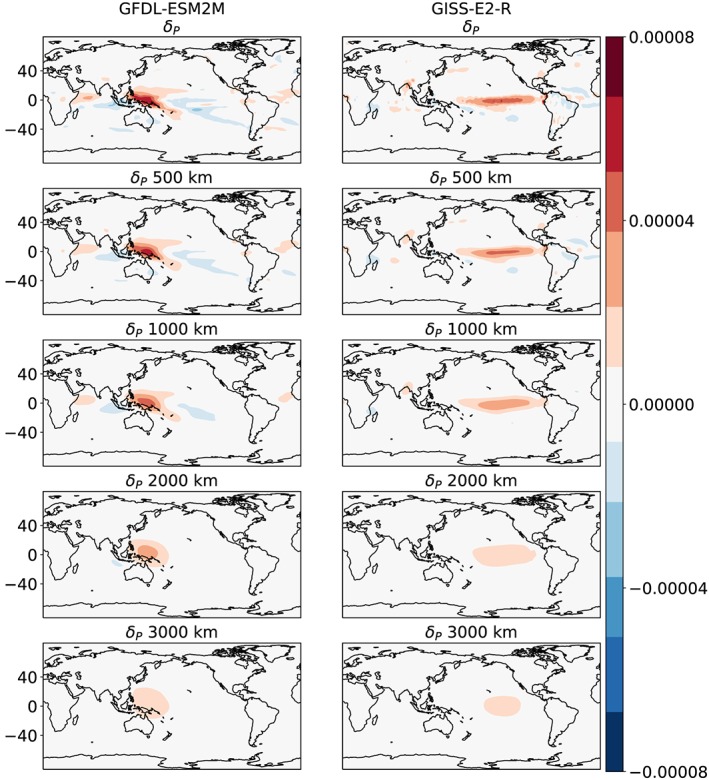
Examples from two Coupled Model Intercomparison Project Phase 5(CMIP5) models of the changes in precipitation (*δP* [kg/s/m^2^]) at different spatial scales. The top row shows the native model resolution; the subsequent rows are averaged over a circle centered at each grid point with the given radius indicated in the title. The left column presents the results from the Geophysical Fluid Dynamics Laboratory Earth System Model with Modular Ocean Model 4 component (GFDL‐ESM 2M) model (Dunne et al., 2012), while the right column presents the results from the Goddard Institute for Space Studies E2‐R (GISS‐E2‐R) model (Schmidt et al., 2014).

How do the scales of the water budget (*P* − *E*) closure compare to the scales of changes in precipitation due to climate change? To answer this question, we examine the scales of precipitation changes (*δP*—define as the precipitation in the RCP8.5 simulations minus the precipitation in the historical simulations: Figure 3).

There is a large spread among CMIP5 models in the spatial pattern of precipitation change projections (Knutti & Sedláček, 2013). However, similar to the *P*−*E* (Figure 1), when averaged over increasingly large scale, the pattern of *δP* becomes weaker and almost completely vanish on scales larger than 3,000 km.

**Figure 4:**
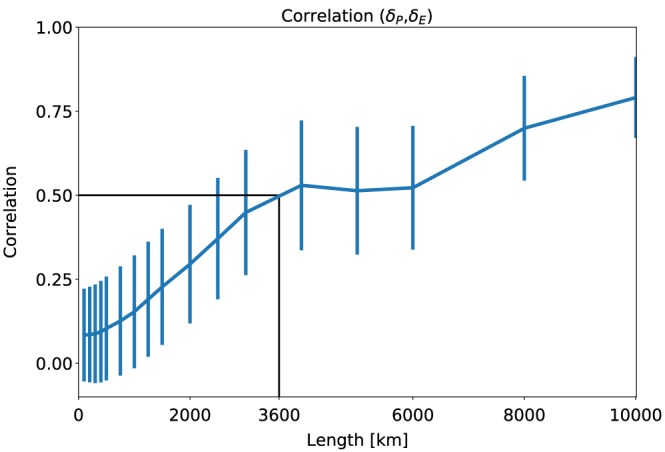
Multimodel mean spatial correlation between changes in precipitation (*δP*) and changes in evaporation (*δE*) between the Coupled Model Intercomparison Project Phase 5 (CMIP5) Representative Concentration Pathway 8.5(RCP8.5) simulations and the historical simulations as a function of the spatial scale of averaging. It was calculated by first averaging *δP* and *δE* over a given length scale (*x* axis) and then calculating the spatial correlation between them. The vertical lines present the standard deviation of the different models. The black lines mark the length scale at which the correlation crosses the 0.5 value.

Figure 4 presents the spatial correlation between the changes in precipitation (*δP*) and changes in evaporation (*δE*) between the RCP8.5 simulations and the historical simulations, averaged at different scales. It demonstrates a monotonic increase in correlation with the spatial scale from ~0.1 at the native resolution (1°×1°) to above 0.8 at 10,000 km resolution. This pattern suggests that indeed at small spatial scales, *δP* is not similar to *δE*, and changes in div (*q*_*v*_) dominate (equation (1)). However, once averaged over larger spatial scales, the role of div (*q*_*v*_) in determining precipitation changes decreases. The correlation between *δP* and *δE* becomes larger than 0.5 at ~3,600 km, comparable to the scale needed to close the water budget to within less than 10% (Figure 2). A similar trend of correlation between *δP* and changes in the atmospheric radiative budget was shown by Muller and O'Gorman (2011). In addition, it was recently shown that the tropics reach radiative convective equilibrium on scales of ~5,000 km (Jakob et al., 2019). Some similarities between the scales of constraining precipitation changes due to the water and energy budget are expected due to their coupling. However, these potential connections should be investigated in depth in future work.

## 4 Summary

With a changing climate, precipitation will also change. On a global scale, those changes are expected to be relatively small due to small changes in evaporation (recalling that in steady‐state changes in precipitation and evaporation are constrained by the energy budget of the atmosphere‐surface system; Allen & Ingram, 2002; O'Gorman et al., 2012). However, local precipitation changes are not well constrained by changes in evaporation, as local changes in water vapor convergence (due to circulation changes or water vapor amount changes) can contribute significantly. We propose that this simple argument suggests that there must exist a breakdown scale of the water budget constraint on precipitation—*L*_*WB*_. Below *L*_*WB*_ the system is “free,” and the magnitude of precipitation changes could be much larger than the evaporation changes (the dynamic contribution can dominate; Allen & Ingram, 2002; Chadwick et al., 2013; Ma & Xie, 2013; O'Gorman, 2015; Pfahl et al., 2017; Sato et al., 2017; Weller et al., 2019). However, above *L*_*WB*_, the precipitation changes are limited by the evaporation changes.

Using two different reanalysis data sets, 39 CMIP5 models and additional global simulations, we show that *L*_*WB*_ depends on the degree to which the water budget is closed, but has a similar behavior and magnitude between the different models and reanalysis data sets. If required that the water budget is closed to <10% (allowing small divergence term contributions to precipitation changes), *L*_*WB*_ ≈ 3,500–4,000 km, below which changes in precipitation are not constrained by the small changes in evaporation. This scale is not expected to significantly change under global warming (Figure 2).

Using CMIP5 RCP8.5 and historical simulations, we show that changes in precipitation are not well correlated with changes in evaporation at spatial scales smaller than *L*_*WB*_(10%), suggesting a large contribution from changes in water vapor divergence. However, once averaged over larger scales, the correlation monotonically increases up to ~0.8 when averaged over 10,000 km (Figure 4). The correlation between *δP* and *δE* crosses the 0.5 level for spatial scale of ~3,600 km, similar to *L*_*WB*_(10%). The increase in correlation suggests that on scales >3,600 km, changes in precipitation are no longer dominated by changes in the divergence term. The fundamental argument presented here could be used to understand and predict spatial scales of future precipitation changes, which could have a significant impact on society. For example, it demonstrates that changes in precipitation on the continental scale could be much larger and less constrained than the global mean change.

## Supporting information



Supporting Information S1
