# Supplementary material for: Analysis of the Atmospheric Water Budget for Elucidating the Spatial Scale of Precipitation Changes Under Climate Change
Source: Geophys Res Lett. 2019 Sep 2;46(17-18):10504–11. doi: 10.1029/2019GL084173 (PMC6853411; doi:10.1029/2019GL084173)
Supplement: Supplementary file 1 — Supporting Information S1 [file GRL-46-10504-s001.pdf]

Supporting information of:

# Analysis of the atmospheric water budget for elucidating the spatial scale of precipitation changes under climate change

**Guy Dagan[1], Philip Stier[1] and Duncan Watson-Parris[1]**

[1] Atmospheric, Oceanic and Planetary Physics, Department of Physics, University of Oxford, UK

E-mail: guy.dagan@physics.ox.ac.uk

**Content:**

Fig. S1

Table S1

The supplementary figure S1 presents the mean scale at which the water budget is locally closed ($L_{WB}$) for different degrees of closure for CMIP5 historical simulation. This figure is similar to Fig. 2 in the main text but includes separate calculations for the ocean and land and also for higher latitudes than 40$^o$. It shows that in tropical regions (between -40$^o$ to 40$^o$), $L_{WB}$ is larger over land than over the ocean, indicating that locally the water budget is closed only on larger scales, probably due to net advection of water from ocean to land. Figure S1 also demonstrates that including latitudes higher than 40$^o$ requires larger scales of averaging due to the net advection of water form low to high latitudes. Inclusion of the high latitudes requires very large (almost global) spatial scales to close the water budget to a strict level (2.5-5%), hence these calculations are not presented in Fig. S1. No significant difference (as compare to the spread between the different models) between land and ocean is seen in the global calculations.

In addition, this file contains a table of all the different CMIP5 models used in this study.

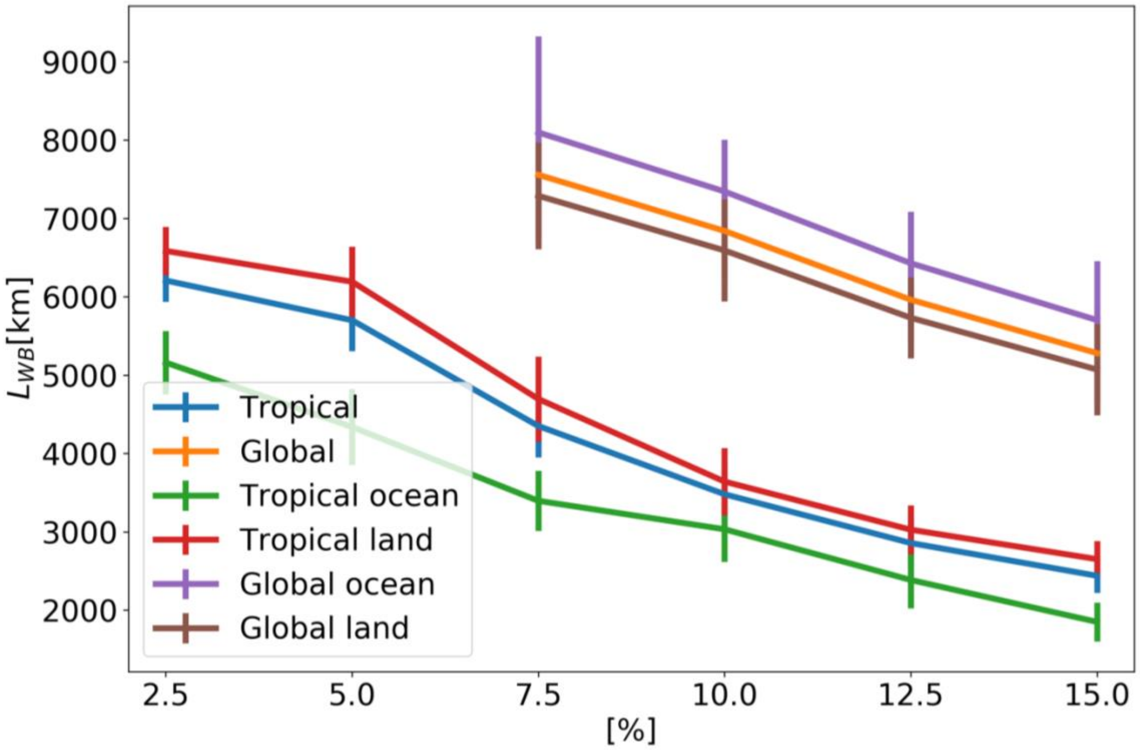


**Figure S1. The spatial scale for local water budget closure ($L_{WB}$ – for which precipitation roughly equals evaporation) as a function of the degree of closure (i.e. the level of similarity between P and E) from the CMIP5 historical simulations. This figure is similar to figure 2 in the main text but present separately calculations for land and ocean and for higher latitude than 40º. The vertical lines represents the standard deviation of the 39 different models.**

**Table S1. List of the 39 CMIP5 models analyzed in this study.**

| Model | Modelling center |
|---|---|
| ACCESS1-0 | Commonwealth Scientific and Industrial Research Organization (CSIRO) and Bureau of Meteorology (BOM), Australia |
| ACCESS1-3 | Commonwealth Scientific and Industrial Research Organization (CSIRO) and Bureau of Meteorology (BOM), Australia |
| BCC-CSM-1 | Beijing Climate Center, China Meteorological Administration |
| BCC-CSM 1-1-3 | Beijing Climate Center, China Meteorological Administration |
| BNU-ESM | Beijing Normal University, Beijing, China |
| CanESM2 | Canadian Centre for Climate Modeling and Analysis |
| CCSM4 | National Center for Atmospheric Research |
| CESM1-BGC | National Center for Atmospheric Research |
| CESM1-CAM5-1-FV2 | National Center for Atmospheric Research |
| CESM1- CAM5 | National Center for Atmospheric Research |
| CMCC-CESM | Centro Euro-Mediterraneo sui Cambiamenti Climatici |
| CMCC-CM | Centro Euro-Mediterraneo sui Cambiamenti Climatici |

| | |
|---|---|
| CMCC-CM5 | Centro Euro-Mediterraneo sui Cambiamenti Climatici |
| CNRM-CM5 | Centre National de Recherches Meteorologiques |
| CSIRO-Mk3-6-0 | Commonwealth Scientific and Industrial Research Organization in collaboration with Queensland Climate Change Centre of Excellence |
| FGOALS-g2 | LASG, Institute of Atmospheric Physics, Chinese Academy of Sciences and CESS, Tsinghua University |
| FIO-ESM | The First Institute of Oceanography |
| GFDL-CM3 | NOAA Geophysical Fluid Dynamics Laboratory |
| GFDL-ESM2G | NOAA Geophysical Fluid Dynamics Laboratory |
| GFDL-ESM2M | NOAA Geophysical Fluid Dynamics Laboratory |
| GISS-E2-H | NASA Goddard Institute for Space Studies |
| GISS-E2-H-CC | NASA Goddard Institute for Space Studies |
| GISS-E2-R | NASA Goddard Institute for Space Studies |
| GISS-E2-R-CC | NASA Goddard Institute for Space Studies |
| HadGEM2-CC | Met Office Hadley Centre |
| HadGEM2-ES | Met Office Hadley Centre |
| INMCM4 | Institute for Numerical Mathematics |
| IPSL-CM5A-LR | Institut Pierre-Simon Laplace |
| IPSL-CM5A-MR | Institut Pierre-Simon Laplace |
| IPSL-CM5B-LR | Institut Pierre-Simon Laplace |
| MIROC5 | Atmosphere and Ocean Research Institute (The University of Tokyo), National Institute for Environmental Studies, and Japan Agency for Marine-Earth Science and Technology |
| MIROC5-ESM | Atmosphere and Ocean Research Institute (The University of Tokyo), National Institute for Environmental Studies, and Japan Agency for Marine-Earth Science and Technology |
| MIROC5-ESM-CHEM | Atmosphere and Ocean Research Institute (The University of Tokyo), National Institute for Environmental Studies, and Japan Agency for Marine-Earth Science and Technology |
| MPI-ESM-LR | Max Planck Institute for Meteorology |
| MPI-ESM-MR | Max Planck Institute for Meteorology |
| MRI-CGCM3 | Meteorological Research Institute |
| MRI-ESM1 | Meteorological Research Institute |
| NorESM1-M | Norwegian Climate Centre |
| NorESM1-ME | Norwegian Climate Centre |
